# Sensing of *Escherichia coli* and LPS by mammary epithelial cells is modulated by O-antigen chain and CD14

**DOI:** 10.1371/journal.pone.0202664

**Published:** 2018-08-24

**Authors:** Mégane Védrine, Camille Berthault, Cindy Leroux, Maryline Répérant-Ferter, Christophe Gitton, Sarah Barbey, Pascal Rainard, Florence B. Gilbert, Pierre Germon

**Affiliations:** 1 ISP UMR 1282, INRA, Université François Rabelais de Tours, Nouzilly, France; 2 UE 0326 Domaine Expérimental du Pin-Au-Haras, INRA, Le-Pin-Au-Haras, France; University of Illinois, UNITED STATES

## Abstract

*Escherichia coli* is one of the major pathogens causing mastitis in dairy cattle. Yet, the factors which mediate the ability for *E*. *coli* to develop in the bovine mammary gland remain poorly elucidated. In a mouse model, infections induced by the reference mastitis *E*. *coli* P4 showed a strong colonisation of the mammary gland, while this strain had a low stimulating power on cells of the PS bovine mammary epithelial cell line. In order to understand if such a reduced response contributes to the severity of infection, a library of random mutants of P4 strain was screened to identify mutants inducing stronger response of PS cells. Among hyper-stimulating mutants, six were altered in genes involved in biosynthesis of lipopolysaccharide (LPS) and had lost their O-polysaccharide region, suggesting that the presence of O-antigen impairs the response of PS cells to LPS. Using purified smooth (S) and rough (R) fractions of LPS, we showed that the R-LPS fraction induced a stronger response from PS cells than the smooth LPS fraction. Biological activity of the S-LPS fraction could be restored by the addition of recombinant bovine CD14 (rbCD14), indicating a crucial role of CD14 in the recognition of S-LPS by Mammary Epithelial Cells (MEC). When S-LPS and R-LPS were injected in udder quarters of healthy lactating cows, an inflammation developed in all infused quarters, but the S-LPS induced a more intense pro-inflammatory response, possibly in relation to sizeable concentrations of CD14 in milk. Altogether, our results demonstrate that the O-antigen modulates the pro-inflammatory response of MEC to LPS, that S-LPS and R-LPS trigger different responses of MEC and that these responses depend on the presence of CD14.

## Introduction

Bovine mastitis is defined as an inflammation of the mammary gland in cows. This disease remains an important issue with a major economic impact for the dairy farmers [1, 2]. In most cases, mastitis results from bacterial infections. Among responsible pathogens, *Escherichia coli* is the most common Gram-negative bacterium causing clinical mastitis in cows worldwide.

When *E*. *coli* bacteria enter the udder and multiply in milk, they encounter host cells, in particular mammary epithelial cells. Bovine Mammary Epithelial Cells (MEC) play a major role in the innate immune system of the bovine mammary gland. They respond to Microbe-Associated Molecular Patterns (MAMPs), which can be recognized by specific and highly conserved Patterns Recognition Receptors (PRRs) [3, 4]. The MEC respond by secreting chemokines (such as CXCL8, CCL20) and pro-inflammatory cytokines (TNF-α, IL-6, IL-1β) necessary for the recruitment of neutrophils in the mammary gland [5–7]. This massive influx of neutrophils will resolve the infection through sequestration and killing of the invading bacteria. Lipopolysaccharide (LPS), the major outer membrane component of Gram-negative bacteria, is also the major MAMP of *E*. *coli* and an important bacterial virulence factor in coliform mastitis [8–10]. The complex of toll-like receptor 4 (TLR4) and the myeloid differentiation protein 2 (MD-2) constitutes the cellular signalling system that allows the recognition of LPS [11].

LPS is composed of three distinct regions: the lipid A, the core oligosaccharide, further divided into outer and inner part and the O-antigen polysaccharide. LPS from the bacterial cell wall is composed of two forms of LPS: smooth- and rough-LPS [12–14]. Smooth-LPS (S-LPS) is composed, in addition to lipid A, of a core oligosaccharide and the O-antigen polysaccharide, which is made up of repeated oligosaccharide units. Many clinically relevant Gram-negative bacteria express this type of LPS. In contrast, rough (R) strains express a core oligosaccharide but lack an O-antigen. More than 180 different O-antigens, defining different serogroups, have been described for *E*. *coli* [15]. Many roles have been attributed to O-antigen in the literature. The LPS O-antigen moiety of uropathogenic *E*. *coli* (UPEC) was associated with the magnitude of cytokine production such as IL-6 [16]. LPS can activate the classical and the alternative complement pathways and the O-polysaccharide moiety of LPS can sterically hinder the access of complement components to the bacterial membrane [17, 18]. Moreover, deletion of the *waaL* gene, which encodes O-antigen ligase, an enzyme responsible for the attachment of O-antigen to lipid A-core oligosaccharide, reduced the bacterial virulence in ducklings [19].

*E*. *coli* P4, originally isolated from a case of clinical bovine mastitis has been used in a number of studies and has been accepted as a reference mastitis strain. This strain is of serotype O32, and intra-mammary inoculation of this strain in cows reproduces the disease [20]. *In vivo*, in a murine model, strain P4 induced more severe mastitis with higher bacterial loads, higher neutrophil counts and higher secretion of IL-6 and MIP2 in comparison to other strains. Yet, this strain, which is resistant to bovine complement mediated killing and is able to grow in milk, has a low stimulating power on the PS cell line, a bovine MEC cell line [21]. One hypothesis was that P4 could divert the sensing capacity of the MEC, which would delay the immune response of the udder. This delay in the recognition of the pathogen could permit the multiplication of bacteria and lead to more severe infections [22]. To confirm this hypothesis, the determinants responsible for the weak stimulation induced by *E*. *coli* P4 will need to be identified. In our study, a library of random mutants of P4 strain was constructed and screened to identify mutants that have a higher ability to stimulate MEC. Among hyper-stimulating mutants, we have identified six mutants impaired in the biogenesis of the LPS. Their biological properties were characterized. Since four of the mutants expressed only R-LPS, we performed the isolation of R-fraction and S-fraction from the natural *E*. *coli* P4 LPS. To better understand if these two forms of LPS could induce different responses from the mammary gland or the epithelium, we investigated their impact on the induction of the innate immune response by MEC *in vitro* and after intramammary infusion *in vivo*.

## Materials and methods

### Ethic statement

Animal experiments were conducted at the French National Institute for Agricultural Research (INRA) of Le Pin-au-Haras (Normandie, France) with the approval of the ethics committee « Comité Rennais d’Ethique en matière d’Expérimentation Animale » (agreements No 2016082518447444-V2). Animal studies were compliant with all applicable provisions established by the European directive 2010/63/UE. Cows were housed in a free-stall barn and milked twice daily. Water was provided ad libitum. Cows were allowed continuous ad libitum intake of a lactation Total Mixed Ration. Enrichment consisted in free access to a resting area and to a cow brush. Analgesics were not used in order to avoid any interference with inflammatory parameters measured during this study. To avoid unnecessary suffering and contain the inflammatory reaction, the amount of LPS used (1 μg) to trigger intra-mammary inflammation was significantly reduced compared to other protocols described in the literature.

### *E*. *coli* strain

Strain P4 (serotype O32:H37) is a prototypical *E*. *coli* mastitis strain isolated from a bovine case of clinical mastitis [20]. Bacteria were grown routinely in Brain Heart Infusion broth (BHI) with or without shaking or on solid tryptic soy agar (TSA) medium at 37°C. When necessary, the BHI medium was supplemented with kanamycin at 25 μg/mL. Strains are stored at -80°C, in BHI medium containing 15% glycerol.

### Culture of the PS bovine mammary epithelial cell line

PS cells are a bovine mammary epithelial cell line obtained in our laboratory from secretory parenchyma [23]. Cells were cultured at 37°C in 5% CO_2_ in Advanced DMEM/F-12 medium (Gibco) containing 4 ng/mL of hydrocortisone (Gibco), 2mM of glutamine (Gibco), 20 mM of HEPES (Biowhittaker), IGF-I (Insulin-like Growth Factor; 10 ng/mL; Peprotech), FGF (Fibroblast Growth Factor; 5 ng/mL; Peprotech) and EGF (Epidermal Growth Factor; 5 ng/mL; Sigma).

### Random transposome mutagenesis of *E*. *coli* P4

*E*. *coli* P4 was subjected to transposome mutagenesis using EZ-Tn5^™^<KAN-2>Tnp Transposome^™^ kit (Epicentre) according to the procedure supplied by the manufacturer. Briefly, one microliter of EZ-Tn5 Tnp transposome complex containing 20 ng transposome was used to electroporate *E*. *coli* P4 cells previously rendered competent [24]. The electroporated cells were recovered by adding SOC medium to the electroporation cuvette to 1 mL final volume immediately after electroporation. Cells were incubated on a 37°C shaker for 1 hour. Transformants were obtained by plating on LB agar containing 50 μg/mL kanamycin and incubating at 37°C for 2 days. The kanamycin resistant transformants were individually picked and inoculated in 96-well microplates in LB medium containing 15% glycerol before being stored at -80°C. Stimulation assays on PS cells were performed and mutants with a higher capacity to stimulate cells were selected.

### Determination of transposome insertion sites

To determine the specific transposome insertion site in each mutant, DNA was isolated using the Wizard Genomic DNA Purification Kit (Promega), and Tn5 insertion sites within the genome were analysed by modified arbitrary polymerase chain reaction (PCR) [25]. In the first PCR, primers ARB1 (5’-GGCCACGCGTCGACTAGTACNNNNNNNNNNGACTG-3’) and ARB2 (5’-GGCCACGCGTCGACTAGTAC-3’) were used with PCR cycles as follows: initial denaturation for 5 min at 95°C; 6 rounds of 95°C for 30 s, 30°C for 30 s, and 72°C for 1 min and 30 s and 30 rounds of 95°C for 30 s, 45°C for 30 s, 72°C for 2 min. The reactions for the second round of PCR were performed using primers ARB2, (5’-GGCCACGCGTCGACTAGTAC-3’) and ARB3 (5’-GGCCACGCGTCGACTAGTACNNNNNNNNNNGAATG-3’) with reactions conditions as follows: initial denaturation of 5 min at 95°C and 30 rounds of 95°C for 30 s, 52°C for 30 s, 72°C for 2 min. The PCR products were purified from an agarose gel using the Nucleospin^®^ Gel and PCR Clean-up kit (Macherey-Nagel) as described by the manufacturer. The purified PCR products were sequenced using the primer KAN-2 FP-1 (5′—ACCTACAACAAAGCTCTCATCAACC—3′) by Genewiz (Takeley, United Kingdom).

### Stimulation of PS cells with live bacteria and purified bacterial agonists

Stimulation assays of bovine MEC were performed as described previously [21, 26]. Briefly, PS cells were seeded at a concentration of 10^5^ cells/mL in 6-well or 96-well plates in GM medium and allowed to grow to confluence. Sixteen hours before stimulation, stimulating medium, i.e. growth medium without growth factors was added. Cells were stimulated with either live bacteria at a multiplicity of infection (MOI) of 1 or with 250 ng/mL of purified LPS diluted in fresh stimulation medium. When required, 0.5 μg/mL of recombinant bovine soluble CD14 was added to the medium. Recombinant bovine CD14 was purified as described previously [21]. After 8 hours of stimulation with live bacteria or 5 hours with purified LPS, supernatants were collected and stored at -20°C. The CXCL8 production by PS cells was quantified by the enzyme-linked immunosorbent assay (ELISA) with reference to standard as described previously [27]. Samples were analysed in duplicate.

### Sodium dodecyl sulfate-polyacrylamide gel electrophoresis (SDS/PAGE) analysis of LPS from whole-cell lysates

LPS phenotypes of *E*. *coli* P4 or mutants were analysed using the whole-cell lysates method developed by Hitchcock and Brown [28]. Bacteria were first seeded in BHI medium, with kanamycin at 25 μg/mL when necessary, for 8 hours at 37°C without agitation. These precultures were diluted 100-fold in DMEM/F-12 medium (Gibco) and incubated for 16 hours at 37°C without agitation. Bacterial cultures were then centrifuged at 5,900 x *g* for 10 min at 4°C. The pellet was suspended in 0.0625M Tris buffer (pH 6.8) to obtain OD600 value of 10. Next, one fourth of the latter volume of 0.0625 M Tris buffer (pH 6.8) containing 8% SDS was added. Samples were heated at 100°C for 10 min, and the lysates were cooled to 55°C. Ten microliters of proteinase K (Sigma– 10mg/mL) was added per 25 μl of lysate, and the samples were incubated at 55°C for 2 hours and then kept overnight at 20°C. Sample buffer containing 2% of glycerol and bromophenol blue was added. Samples were heated at 100°C for 10 min and loaded on 14 x 14 cm acrylamide gels and electrophoresis was performed at 30 mA for 5 hours at 17°C. The running (15% acrylamide/bis acrylamide (29–1)) and stacking gels (4%) were prepared as described by Laemmli [29]. Routinely, after addition of the sample buffer, 20 μl of a sample were applied per slot. The Precision Plus Protein^™^ All Blue Standards marker (Biorad) were used. LPS molecules were revealed by silver staining after periodate oxidation as described previously [30].

### Stimulation of TLR2 and TLR4 receptors in transfected HEK293 cells with live bacteria

HEK293 cell lines, stably expressing human TLR2 (HEK/TLR2) or TLR4 (HEK/TLR4-MD2-CD14) (InvivoGen) were used. Cells were cultivated in DMEM high glucose (Gibco) containing 2 mM of glutamine, 20 mM of HEPES, 10% heat-inactivated fetal bovine serum (Gibco) and 1x HEK-Blue Selection solution (InvivoGen) and grown at 37°C under an atmosphere of 5% CO_2_. For cell-stimulation experiments, HEK cells were seeded at a density of 2.5x10^5^ cells/mL in 96-well plates and grown to 80–90% confluence. Sixteen hours before stimulation, stimulation medium was added. Just before the stimulation fresh stimulation medium was added and cells were incubated for 3 hours at an MOI of 1 with live *E*. *coli*. Supernatants were then removed, cells were washed once with HBSS and stimulation medium containing 100 μg/mL gentamicin was added. Incubation was extended for 5 hours for stimulations with bacteria. Supernatants were then collected and frozen at -20°C until human CXCL8 quantification using the Human CXCL8 Standard ABTS ELISA kit (Peprotech) according to the procedure supplied by the manufacturer.

### Growth of bacteria in LB medium and bovine milk

*E*. *coli* P4 and mutants were precultured at 37°C during 8 hours in BHI, with kanamycin at 25 μg/mL when necessary, then 100-fold dilution were performed in LB medium and incubated at 37°C for 16 hours. Bacteria were pelleted and washed in DPBS without calcium and magnesium (Lonza) and were then used to inoculate sterile spectrophotometer cuvettes (initial theorical optical density at 600 nm, OD600 = 0.04) containing LB medium. Cuvettes were incubated 5 hours at 37°C. The OD600 was measured after 1 hour and then every thirty minutes.

Fresh raw milk was aseptically collected from a healthy cow belonging to the dairy facility of the Experimental Unit of Animal Physiology (UE PAO, INRA Val de Loire). The inflammatory milk was obtained after infusion of LPS in a mammary quarter of a cow from the INRA dairy herd at Le Pin-Au-Haras. *E*. *coli* P4 and mutants were first seeded in BHI medium with kanamycin at 25 μg/mL when necessary, for 16 hours at 37°C. Bacterial cultures were then centrifuged (5 min at 4,500 x *g*) and washed with sterile DPBS without calcium and magnesium. Fifteen milliliters of milk were inoculated with the bacterial suspension at a concentration of 10^4^ cfu/mL, and was immediately dispensed in 1.5 mL microtubes (1.5 mL per tube) and incubated at 37°C. Every hour, CFUs from one microtube were counted by serial dilutions and plating on TSA plates.

### Resistance to bovine complement system

Serum was prepared from blood collected from 5 healthy Holstein dairy cows as described previously [21]. To collect blood, cows were restrained at the feeding fence in order to expose the jugular groove. Ten ml of blood was drawn from the jugular vein in a Venosafe tube and processed as described [21]. *E*. *coli* P4 and mutants were grown in DMEM/F-12 medium with or without kanamycin at 25 μg/mL for 8 hours at 37°C and then diluted 100-fold in DMEM/F-12 medium and incubated for 16 hours at 37°C. Bacteria were adjusted at the concentration of 8.10^3^ cfu/mL in 200 μL of DMEM/F-12 and mixed with either 5% of non-heated or heat-inactivated bovine serum. Survival was determined by plate count after 3 hours of incubation at 37°C without agitation. Assays were performed four times, and the percentage of survival was calculated as the ratio of the number of cells obtained after 3 hours in serum to the number of cells obtained after 3 hours in heat inactivated serum multiplied by 100.

### Resistance to polymyxin B

The susceptibility of *E*. *coli* P4 and mutants to polymyxin B (Sigma) was evaluated. Bacteria were first seeded in BHI medium with or without kanamycin at 25 μg/mL for 8 hours at 37°C. The precultured bacterial suspensions were then diluted 100-fold in Mueller-Hinton (MH) medium and incubated for 16 hours at 37°C. The 96-well plates were prepared with fifty microliters of serial dilutions of polymyxin B from 64 μg/mL to 0.0625 μg/mL in MH medium. An inoculum at a final concentration of 2.10^6^ CFU/mL in MH medium was added into the wells. The plates were incubated at 37°C and read after 24 hours. The minimum inhibitory concentration (MIC) was defined as the lowest concentration of antimicrobial agent at which no visible growth could be detected by OD600 readings.

### Extraction and fractionation of LPS from *E*. *coli* P4

*E*. *coli* P4 was grown in LB medium at 37°C without agitation for 16 hours. After centrifugation and a washing step, the LPS was extracted by the phenol/EDTA/triethylamine (phenol/EDTA/TEA) procedure described by Ridley *et al*.[31]. After extensive dialysis against deionized water, the crude LPS was recovered by ultracentrifugation at 200,000 x *g* for 16 hours at 4°C. The pellet containing the crude LPS was resuspended with 4 mL of PBS and subjected to successive enzymatic treatments to remove contaminants. First, the sample was treated with 50 μL of RNase A (10 mg/mL; Fermentas) and 100 μL of DNase I (1U/μl; Fermentas) at 37°C for 2 hours and then by proteinase K at 0.1 mg/mL (Sigma) at 55°C for 2 hours and finally by pronase at 2mg/mL (Boehringer Mannheim) at 40°C for 2 hours. To address the purity of our LPS preparation, the OD at 260 nm and 280 nm were determined and the putative presence of TLR2 agonists was analysed using HEK/TLR2 cells in presence of HEK-Blue^™^ Detection medium (Invivogen). HEK-Blue^™^ Detection changes to a purple/blue color in the presence of alkaline phosphatase activity. The enzymatic treatments were reproduced until we observed an absence of TLR2 contaminants in the LPS preparation.

Then, the native P4 strain LPS was fractionated by size-excusion chromatography with a Sephacryl S-200 column (Amersham Pharmacia Biotech) at room temperature, in a buffer consisting of 0.15 M NaCl, 0.2% deoxycholate (w/v), 2 mM EDTA, and 10 mM Tris (pH 8.0). The collected fractions were analysed by SDS-PAGE with 14% acrylamide gels and LPS bands were revealed by silver staining as described previously [30]. Fractions containing only S-LPS were pooled, dialyzed against deionized water, concentrated, sterilized by filtration on 0.45 μm and stored on ice at 4°C. The absence of TLR2 agonists was checked using HEK/TLR2. The activation of TLR4 by purified LPS and S-LPS fraction was confirmed using HEK/TLR4 cells in presence of HEK-Blue^™^ Detection. LPS were then quantified by the dosage of the 3-hydroxymyristic acid of lipid A by EndoQuant (Faculté de Médecine, Dijon) [32].

### Preparative electrophoresis and analysis of the R-LPS

Sephacryl-S200 fractions containing mostly R-LPS were dialyzed, concentrated to 1 mL and subjected to preparative electrophoresis in presence of DOC using the model 491 Prep Cell from Bio-Rad. The sample containing R-LPS was loaded onto a 5 cm long, 14% polyacrylamide gel casted in the 37 mm diameter gel tube. The separating gel was formed with 14% acrylamide and 0.8% DOC and the 4% acrylamide stacking gel contained 0.5% DOC. Before application on the gel, bromophenol blue, glycerol and DOC (1% final, w/v) were added to the sample. The running buffer contained 0.2% DOC. The electrophoresis was performed at 12 watts with refrigeration and when the front dye reached the bottom of the gel tube, the elution was performed at 0.4 mL/min using a peristaltic pump and fractions of 6 mL were collected. Then, the fractions were analysed by SDS-PAGE with 14% acrylamide gels and Laemmli sample buffer and LPS bands were revealed by silver staining as described previously [30]. Fractions containing only R-LPS were pooled, dialyzed against deionized water, concentrated, sterilized by filtration on 0.45 μm and stored on ice at 4°C. The absence of TLR2 agonists and the capacity of activation of TLR4 receptor were checked using HEK/TLR2 and HEK/TLR4 cells and the accurate concentration of R-LPS was determined by the dosage of the 3-hydroxymyristic acid of lipid A (EndoQuant, Dijon).

### Transcript analysis by RT-qPCR

MEC responses induced by LPS fractions were analysed by quantitative real-time PCR. Total RNA (100 ng -1μg per reaction) was reverse transcribed to cDNA using iScript^™^ Reverse Transcription Supermix (Biorad) according to manufacturer’s instructions. Primers used in this study are listed in S1 Table. Relative quantities of transcripts of interest were analysed by RT-qPCR as described previously [33]. Briefly, after normalization using the expression of 3 reference genes (ACTB, PPIA, 18S RNA), expression of each gene was calculated relative to the values obtained from unstimulated cells arbitrarily set to 1.

### Bovine intramammary LPS challenge

Six cows were recruited for this experiment. Two cows were in their fourth lactation (217 and 173 days in milk (DIM)), 2 in third (163 and 270 DIM) and 2 in second lactation (318 and 238 DIM). Healthy mammary quarters of the udder were selected based on an absence of detectable bacterial growth and a somatic cell concentration (SCC) lower than 100,000 cells/mL in milk, indicating the absence of inflammation. One day before the experiments, quarter milk samples were taken under aseptic conditions for bacteriological examination by plating 30 μL of milk on sheep blood agar plates and recording bacterial colonies after 24 hours of incubation at 37°C. A 500 μL portion of milk was subjected to a Fossomatic model 90 (Foss Electric, Hillerod, Denmark) to determine the milk SCC.

The intramammary LPS infusion was conducted just after the morning milking of cows. The teats were disinfected with cotton dipped in 70% ethanol. Thereafter, all cows received three intramammary injections of different fractions of LPS namely 1 μg of native-LPS, 1 μg of S-LPS and 1 μg of R-LPS from *E*. *coli* P4 in three different quarters. LPS were diluted in 2 mL of sterile solution of DPBS containing 0.5%(w/v) of BSA (bovine serum albumin solution, Sigma) and infused using a sterile syringe fitted to a 32 mm length cannula. Four of these six cows received 2 mL of a sterile PBS-BSA 0.5% solution as a control in their fourth quarter.

Just before the inoculation of the LPS, milk samples were collected and rectal temperatures were measured. After LPS challenge, rectal temperatures and milk samples were obtained at 4, 8, 12, 24, 48 and 72 hours post-infusion. Aseptically collected milk samples were used for bacteriological analysis. A portion of milk was also collected to determine the SCC measurement in milk samples by using the Fossomatic 90 apparatus. Another portion of milk was harvested and stored frozen (-80°C) for cytokines analysis.

### Cytokines and CD14 quantification in milk from LPS challenged cows

Milk samples were analysed for IL-1β, IL-6, CXCL8, CCL20 concentrations, in addition to the CD14 concentration. IL-1β and IL-6 were quantified by ELISA with bovine IL-1β or IL-6 reagent sets from Thermo Fisher Scientific as suggested by the manufacturer. CXCL8 was measured by ELISA as described previously [27].

The ELISA for bovine CD14 and CCL20 were developed in-house. Recombinant bovine CCL20 (bovine CCL20 ORF minus the signal sequence, with a hexahistidine tag) was expressed with the Drosophila Schneider S2 expression system by transfection with the pMT-PURO plasmid as described [34]. The protein was purified by metal affinity chromatography (Ni-NTA superflow column, Qiagen) as described and used to elicit antibodies in rabbits (Eurogentec, Liège, Belgium). Antibodies to CCL20 were affinity-purified by passage over a column of CCL20 coupled to EAH-Sepharose (GE Healthcare Life Sciences), before removal of antibodies directed against the His-tag by passage over a column of the tag sequence peptide coupled to EAH-Sepharose gel. Part of the antibodies were biotinylated with sNHS-lc-biotin (Interchim, France). A sandwich ELISA was developed by using the purified anti-CCL20 antibodies as capture antibodies (1 μg/mL) and biotinylated anti-CCL20 antibodies (0.5 μg/mL) as detection antibodies. Concentrations of CCL20 in milk samples were determined by reference to the standard curve with purified CCL20 (270 to 25,600 pg/mL).

Recombinant bovine CD14 was purified as described previously [21]. Antibodies production and purification were obtained as described above. A sandwich ELISA was developed by using the purified anti-CD14 antibodies as capture antibodies (1 μg/mL) and biotinylated anti-CD14 antibodies (0.25 μg/mL) as detection antibodies. Concentrations of CD14 in milk samples were determined by reference to the standard curve with purified CD14 (312.5 to 20,000 pg/mL).

### Statistical analysis

Statistical analysis were performed with the StatXact software (version 5.0 –Cytel software). For the *in vitro* analysis, unpaired samples were first compared using the Kruskal and Wallis test followed by Mann and Whitney test. The comparison of paired samples, in *in vivo* study, were done with the Friedman test. A p-value < 0.05 was considered significant.

## Results

### Selection of hyper stimulating mutants of *E*. *coli* P4

**Fig 1:**
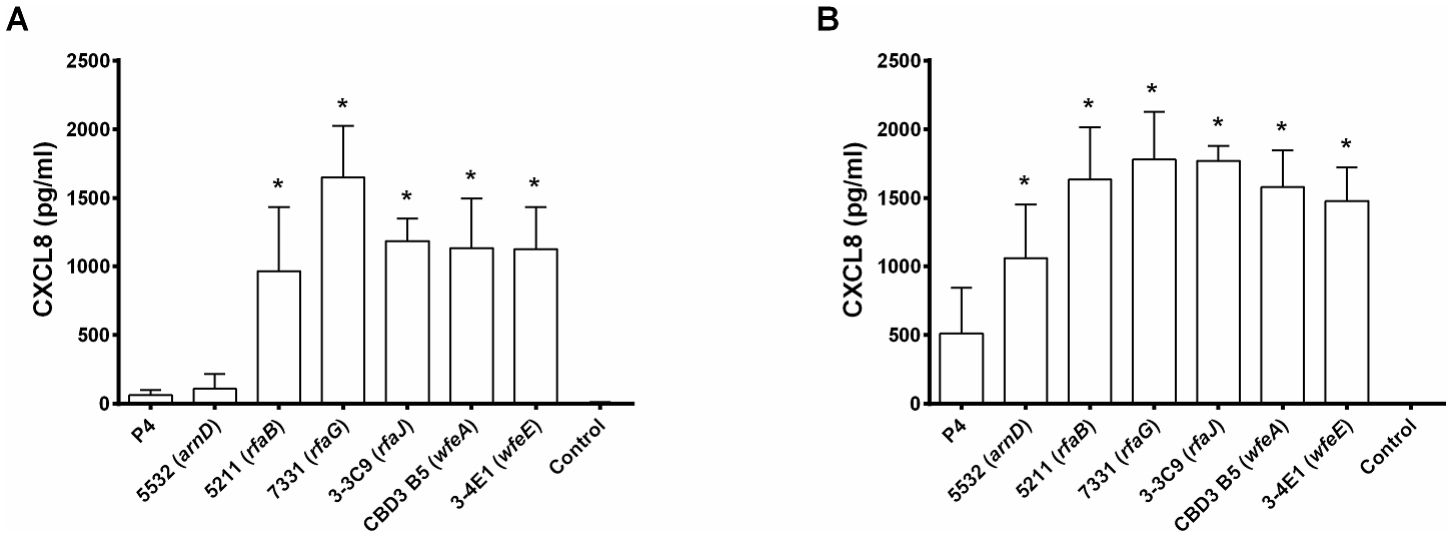
CXCL8 secretion by MEC PS cells upon stimulation with live *E*. *coli* P4 wild type and mutants. PS cells were incubated for 3 hours with the indicated strains at a MOI of 1 in stimulation medium in the absence of rbCD14 (A) or in the presence of 0,5 μg/mL rbCD14 (B). Cells were then washed twice with HBSS and medium with gentamicin was added. Response was analysed by quantification of CXCL8 secretion by ELISA 8 hours after beginning of the experiment. Data presented are mean values and standard deviations obtained from six (- CD14) or at least three (+ CD14) independent experiments with stimulations performed in duplicates. * indicates statistical significance (p < 0,05). p-values were calculated using Mann and Whitney after global comparison using a Kruskal and Wallis test.

We took advantage of our bovine mammary epithelial PS cell line to screen a library of random mutants of strain P4 for clones with increased ability to stimulate MEC. As a mediator of the pro-inflammatory innate immune response of MEC to bacteria, the production of the cytokine CXCL8 in the supernatant of stimulated MEC was used as a read-out. A set of 6 mutants was identified and confirmed to induce increased innate immune response by PS cells (Fig 1). Because our previous studies had indicated that CD14 was important for the recognition of LPS by PS cells [23], the ability of these mutants to stimulate PS cells was also performed in the presence of purified recombinant bovine CD14 (rbCD14) (Fig 1B). While CXCL8 secretion was slightly increased by the presence of rbCD14 for all strains, the higher stimulation observed with mutants compared to WT P4 strain was still observed. Based on this initial screening, these six mutants with a hyper stimulating phenotype were selected and subsequently analysed.

### Identification of genes encoding enzymes involved in LPS biosynthesis

We then used arbitrary-PCR to identify the disrupted gene in the selected mutants. The sequence comparison revealed that all 6 mutants exhibited mutation in genes involved in the synthesis of LPS, either in the core-lipid A region or in the synthesis of O-antigen subunits (Table 1).

**Table 1:** Mutants of *E*. *coli* P4 altered in LPS biogenesis genes.

Mutant	Gene	Protein	Function	Stimulation of MEC: % CXCL8 / P4
**5532**	*arnD*	Undecaprenyl phosphate-α-L-ara4FN deformylase	Lipid A modification	170
**5211**	*rfaB = waaB*	UDP-D-galactose: (glucosyl)lipopolysaccharide-1,6-D-galactosyltransferase	Synthesis of LPS outer-core	1487
**7331**	*rfaG = waaG*	UDP-glucose:(heptosyl)lipopolysaccharide α-1,3-glucosyltransferase	Synthesis of LPS outer-core	2538
**3-3C9**	*rfaJ = waaJ*	UDP-glucose:(glucosyl)lipopolysaccharide α-1,2-glucosyltransferase	Synthesis of LPS core	1825
**CBD3 B5**	*wfeA*	Glycosyltransferase	Synthesis of O antigen	1747
**3-4E1**	*wfeE*	Glycosyltransferase	Synthesis of O antigen	1732

**Fig 2:**
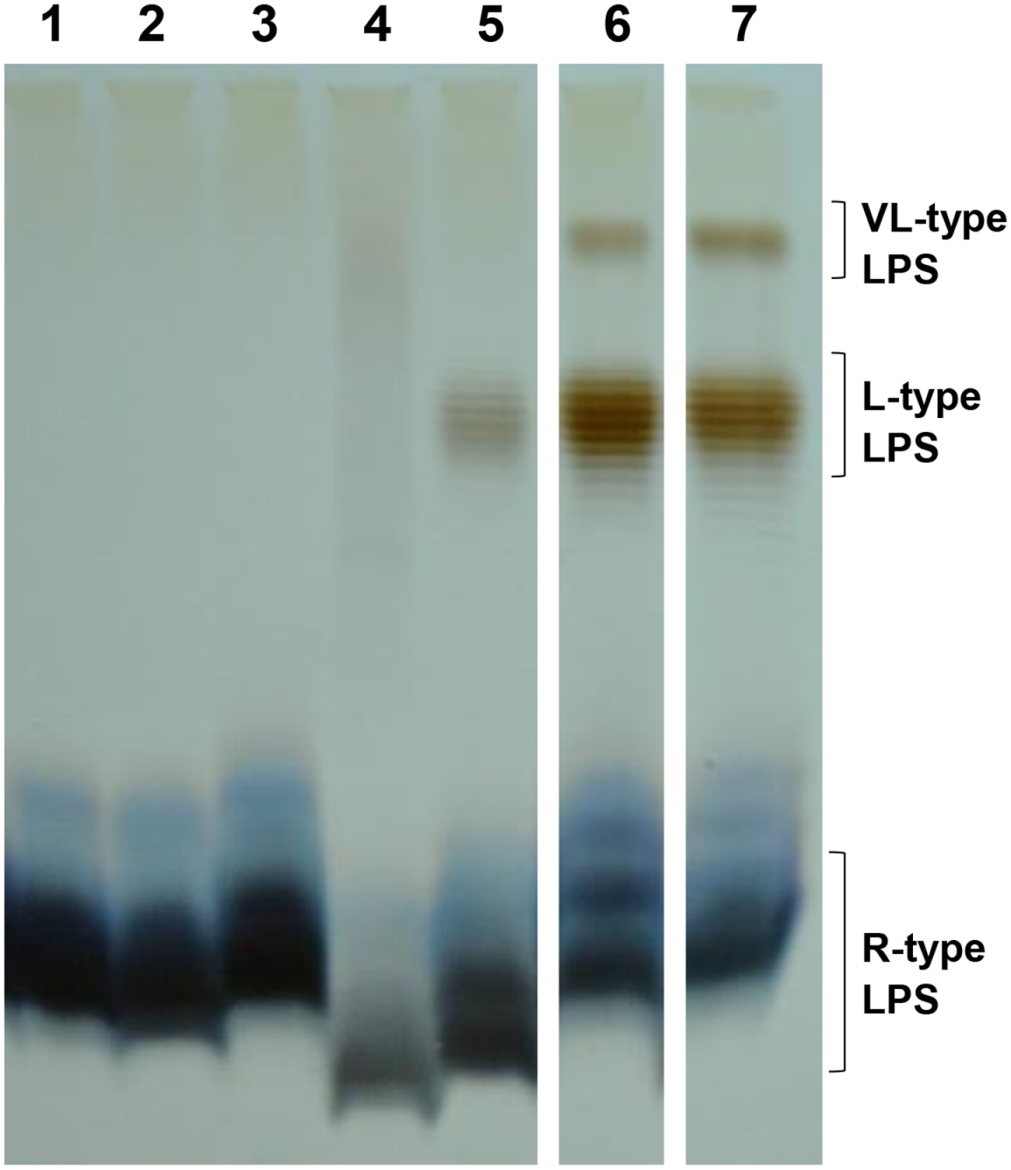
LPS structure analysis of *E*. *coli* P4 wild type and mutants. SDS-PAGE analysis of the LPS fractions from: Lane 1: *wfeE*; Lane 2: *rfaJ*; Lane 3: *wfeA*; Lane 4: *r*f*aG*; Lane 5: *rfaB*; Lane 6: *arnD*; Lane 7: P4 strain wild type. The positions of LPS of various types are indicated on the right. R-type LPS, rough type LPS; L-type LPS, long type LPS; VL-type LPS, very-long type LPS. The 15% polyacrylamide gels were stained with silver nitrate.

Because these genes are involved in LPS biosynthesis, we then analysed the LPS profile of *E*. *coli* P4 and its mutants by SDS/PAGE and silver staining (Fig 2). The WT strain (lane 7) produced LPS with long O antigen chains (L-type) and very long O antigen chains (VL-type). In comparison with the WT strain, mutants produced LPS with reduced amounts of VL and L chains (*rfaB* lane 5) or completely devoid of both chain types (*wfeE* lane 1; *rfaJ* lane 2; *wfeA* lane 3 and *rfaG* lane 4) and/or lipid A modification (lane 2; 4 and 5). Only one mutant, *arnD*, did not show any noticeable alteration compared to the WT strain (lane 6). Compared to the other mutants, the latter induced a reduced pro-inflammatory response of PS cells (Fig 1).

Multiple attempts for plasmid complementation of mutants failed because of the impact of the vector alone on the LPS of bacteria (data not shown). As a surrogate, we compared the pro-inflammatory response triggered by the MG1655 K-12 strain that does not have any O-antigen to that of MG1655 *wbbL*+ in which O-antigen synthesis had been restored [35]. In comparison to MG1655 WT strain, the derived MG1655 *wbbL*+ induced a decreased innate immune response by PS cells (S1 Fig). In presence of rbCD14, while secretion of CXCL8 was slightly increased by the presence of rbCD14 for these two strains, the higher stimulation observed with MG1655 WT compared to MG1655 *wbbL+* strain was still observed.

### Activation of PRRs

Since the mutants displayed important alterations in the structure of their LPS, we hypothesized that their higher stimulating activity was related to a better recognition of their LPS by the toll like receptor (TLR) 4. An HEK293 cell line, which stably expresses human TLR4 with co-receptors CD-14 and MD2, was stimulated and the cytokine CXCL8 was quantified in the supernatant. HEK/TLR4 cells responded in a similar fashion to stimulation with all mutants and WT strain (S2 Fig).

Another hypothesis was that the absence of O antigen could allow a better interaction between others agonists of pattern recognition receptors (PRRs) produced by *E*. *coli*, in particular TLR2 ligands, such as lipoproteins and lipopeptides, which are constituents of the outer membrane of Gram negative bacteria. To test this possibility, we used HEK293 cells expressing human TLR2 and showed that they responded in a similar fashion to stimulation with all strains (S3 Fig).

### Growth of *E*. *coli* in different media

**Fig 3:**
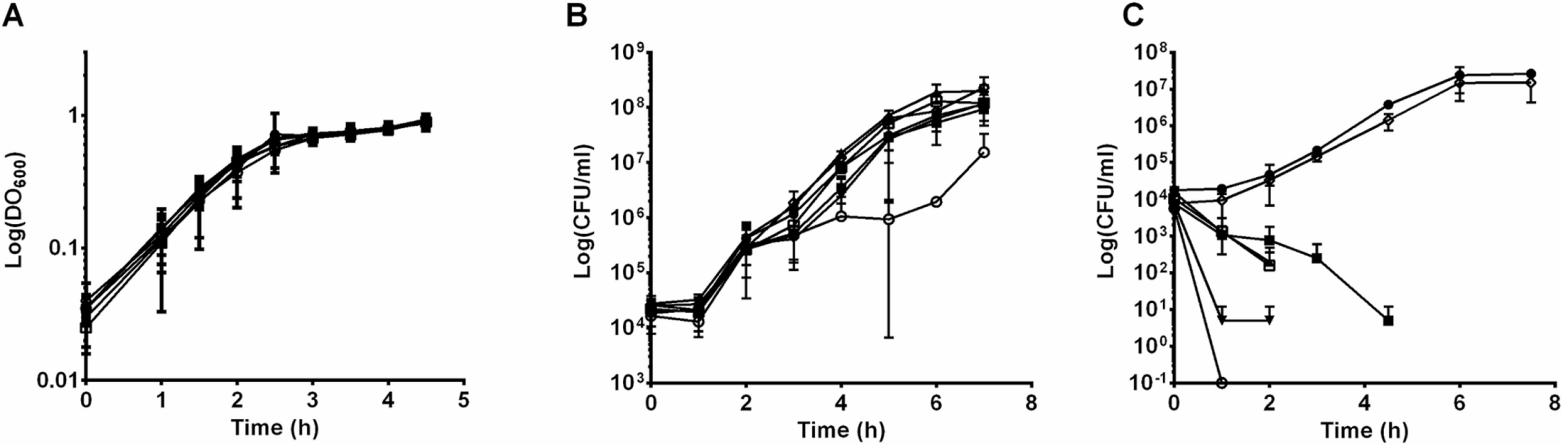
Growth in different media of selected *E*. *coli* P4 wild type and mutants. LB medium (A) was inoculated with an initial optical density at 600 nm of 0.04 of strains P4 strain wild type (●), *arnD* (◊), *rfaB* (▼), *rfaG* (○), *rfaJ* (■), *wfeA* (□), *wfeE* (▲). Growth experiments were performed with measurements of optical density at each time point. Fresh whole milk (B) and inflammatory milk (C) were inoculated with 10^4^ cfu/mL of strains and bacteria were counted at each time point by 10-fold serial dilution and plating on TSA agar plates. Data presented are means of DO_600nm_ or cfu/mL calculated from two independent experiments.

We then demonstrated that mutants and P4 WT strain showed the same growth in rich medium (LB) (Fig 3A). In aseptic fresh milk, except for the *rfaG* mutant that displayed the most important alteration in structure of LPS with modification in lipid A region and an absence of L-type and VL-type O-antigen chains, all strains showed similar growth curves (Fig 3B).

To evaluate if absence of O-antigen had any impact on resistance to higher amounts of complement and mediators of inflammation, we used inflammatory milk obtained from cows infused with LPS. The WT strain was able to grow in these conditions, as was the *arnD* mutant. In comparison, none of the mutants with a rough phenotype was able to survive in inflammatory milk (Fig 3C).

### *E*. *coli* P4 and mutants survival in serum

**Fig 4:**
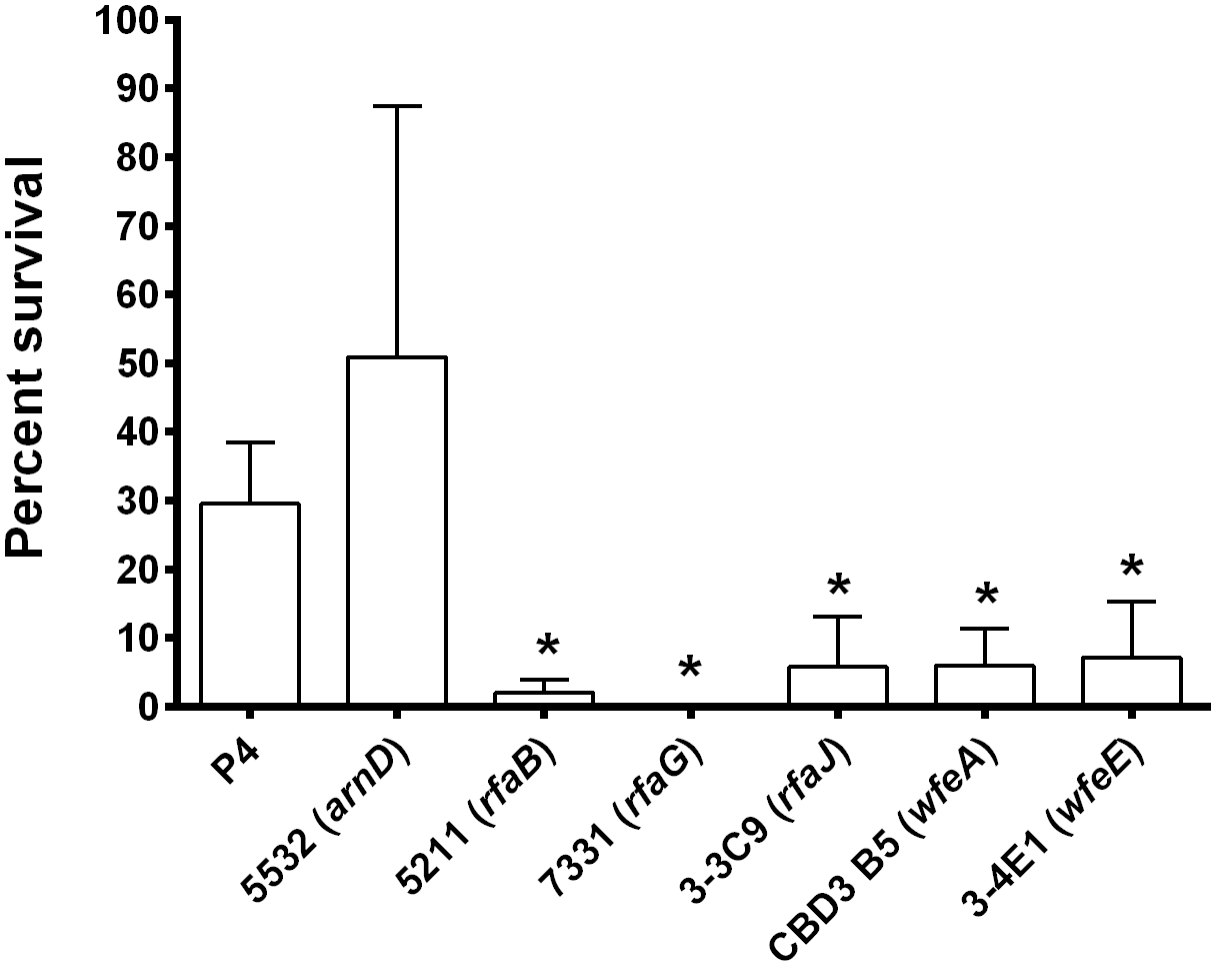
Resistance of *E*. *coli* P4 wild type and mutants to Complement. Strains were incubated, with a starting inoculum of 8.10^3^ cfu/mL, in the presence of whole bovine serum or heat-inactivated serum at concentration of 5% for 3 hours. Data presented are means and standard deviations of the percentage of survival expressed as 100*(number of bacteria obtained after 3 hours in 5% serum / number of bacteria obtained after 3 hours in 5% heat inactivated serum) of four independent experiments. * indicates statistical significance (P < 0.05). p-values were calculated using Mann and Whitney after global comparison using a Kruskal and Wallis test.

Inflammatory milk contains significant amounts of complement that could impede the growth of colonizing *E*. *coli*. Therefore, we measured precisely the resistance of the mutant strains to complement mediated killing. The impact of complement was tested after 3 hours of incubation in the presence of heat-inactivated serum or non-heated serum at a concentration of 5% (v/v) (Fig 4). WT strain (30% viability) and the *arnD* mutant (51%) were rather resistant to complement while *rfaB* (2%), *rfaJ* (6%), *wfeA* (6%) and *wfeE* (7%) were more susceptible to complement. The *rfaG* mutant was unable to survive at all in the presence of non-heated serum.

### The LPS O-antigen influences polymyxin B resistance

We used polymyxin B as a surrogate of antimicrobial peptides. Polymyxin B is a cationic molecule that interacts with high affinity with the negatively charged phosphate groups of the lipid A moiety in LPS. Binding of polymyxin B to lipid A results in a destabilisation and disruption of the bacterial outer membrane, resulting in cytoplasmic leakage and cell death. To test if LPS modification could have an impact on resistance of mutants to polymyxin B, WT strain and its mutants were assayed for polymyxin B resistance by determination of the minimum inhibitory concentration (MIC) by broth microdilution tests. The WT strain was resistant to polymyxin B (MIC = 8 μg/mL), almost to the same level as were the *arnD*, *rfaJ*, *wfeE* and *wfeA* mutants (MIC ≥ 4μg/mL). Mutants *rfaB* and *rfaG* (MIC = 2μg/mL) showed the highest susceptibility to polymyxin B (S4 Fig).

### Isolation and fractionation of *E*. *coli* P4 LPS

**Fig 5:**
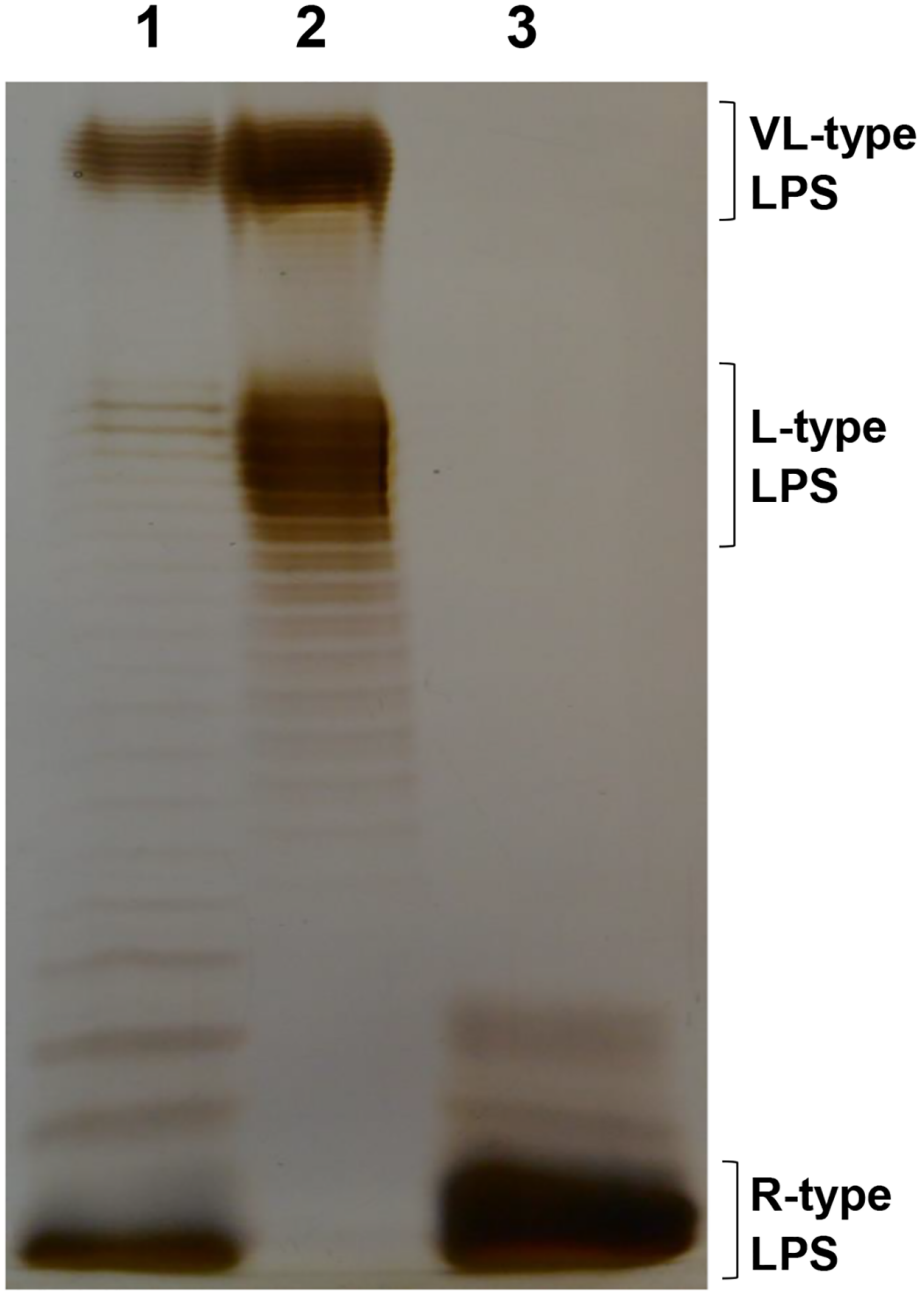
LPS structure analysis from purified LPS fractions from *E*. *coli* P4. SDS/PAGE analysis of isolated LPS fractions from *E*. *coli* P4. Lane: 1, native LPS; Lane 2: smooth LPS, S-LPS fraction; Lane 3: rough LPS, R-LPS fraction. The positions of LPS of various types are indicated on the right. R-type LPS, rough type LPS; L-type LPS, long type LPS; VL-type LPS, very-long type LPS. The 15% polyacrylamide gels were stained with silver nitrate.

The above results suggest that the absence of O-antigen induces a higher ability to stimulate MEC. To confirm this hypothesis, LPS of *E*. *coli* P4 was extracted by the phenol/EDTA/TEA procedure. S-LPS was then further purified by size exclusion chromatography while fractions containing R-LPS with no or only short O-antigen chains were obtained by preparative electrophoresis. Purified S- and R-LPS were examined with SDS-PAGE and silver staining (Fig 5). As expected, LPS purified from P4 strain (lane 1) showed L-type and VL-type LPS along with LPS without O polysaccharide (lane 1). Instead, S-LPS just possesses LPS with L-type and VL-type LPS (lane 2) and R-LPS is free of O polysaccharide (lane 3).

### Activation of MEC by S and R forms of *E*. *coli* LPS

**Fig 6:**
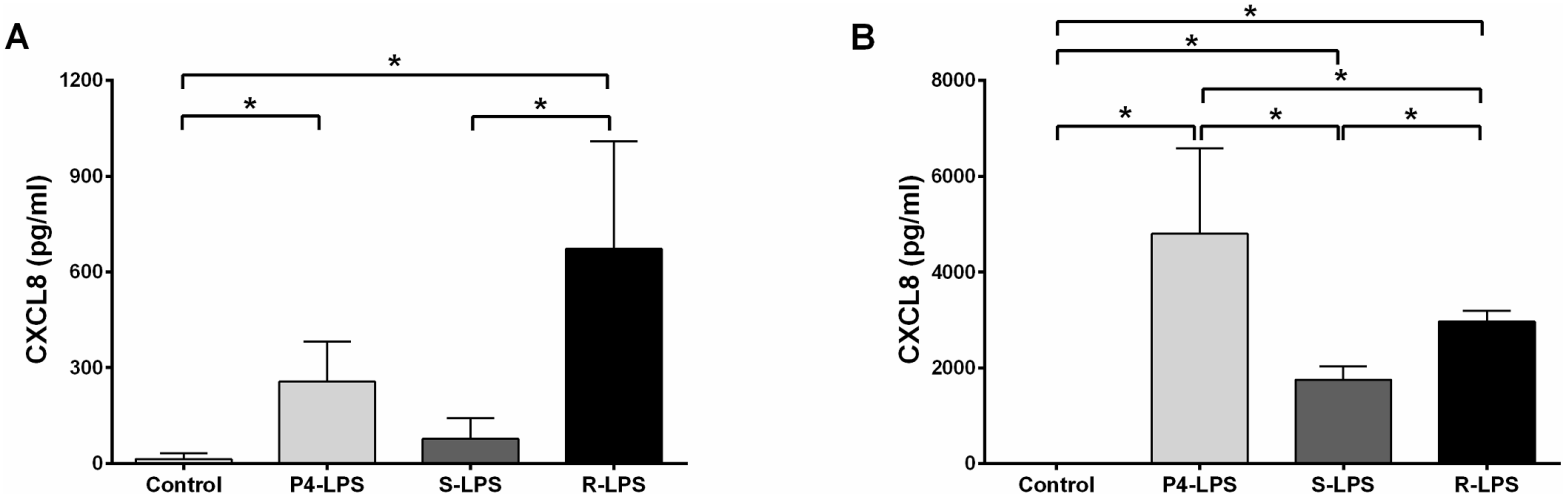
CXCL8 synthesis by PS cells in response to different purified LPS-forms. PS cells were incubated for 3 hours with 250 ng/mL of native P4 strain LPS (light grey bars); S-LPS (dark grey bars) or R-LPS (dark bars) in stimulation medium in the absence of rbCD14 (A) or in the presence of 0.5 μg/mL rbCD14 (B). Cells were then washed twice with HBSS and medium with gentamicin was added. Response was analysed by quantification of CXCL8 secretion by ELISA in supernatants collected 5 hours after beginning of the stimulation. Data presented are mean values and standard deviations obtained from three independent experiments. * indicates statistical significance (p < 0,05). p-values were calculated using Mann and Whitney after global comparison using a Kruskal and Wallis test.

The possible contribution of minor protein contamination in LPS samples to the observed responses was excluded, as activation of a HEK/TLR2 cell line by the LPS samples was negligible in the range of LPS concentrations tested (S5 Fig). To ensure that similar quantities of LPS molecules were used in the different assays, LPS fractions were quantified by dosage of the 3-hydroxymyristic acid of lipid A. The isolated fractions derived from the LPS mixture were then analysed with respect to their biologic activity on PS cells. The production of the cytokine CXCL8 was determined in the supernatant of stimulated PS cells. Without rbCD14, responses of MEC to the purified natural LPS and R-LPS were significantly increased compared to the control. By contrast, S-LPS induced a stronger but not significantly different response compared to the control (Fig 6A). In the presence of rbCD14, secretion of CXCL8 by MEC were significantly increased with all LPS fractions compared to the control. R-LPS was still more efficient to trigger a response by PS cells in comparison to S-LPS (Fig 6B). As a summary, S-LPS was the least potent in the stimulation of the innate response of MEC while R-LPS and natural LPS triggered the highest responses in absence or in presence of rbCD14 respectively.

**Fig 7:**
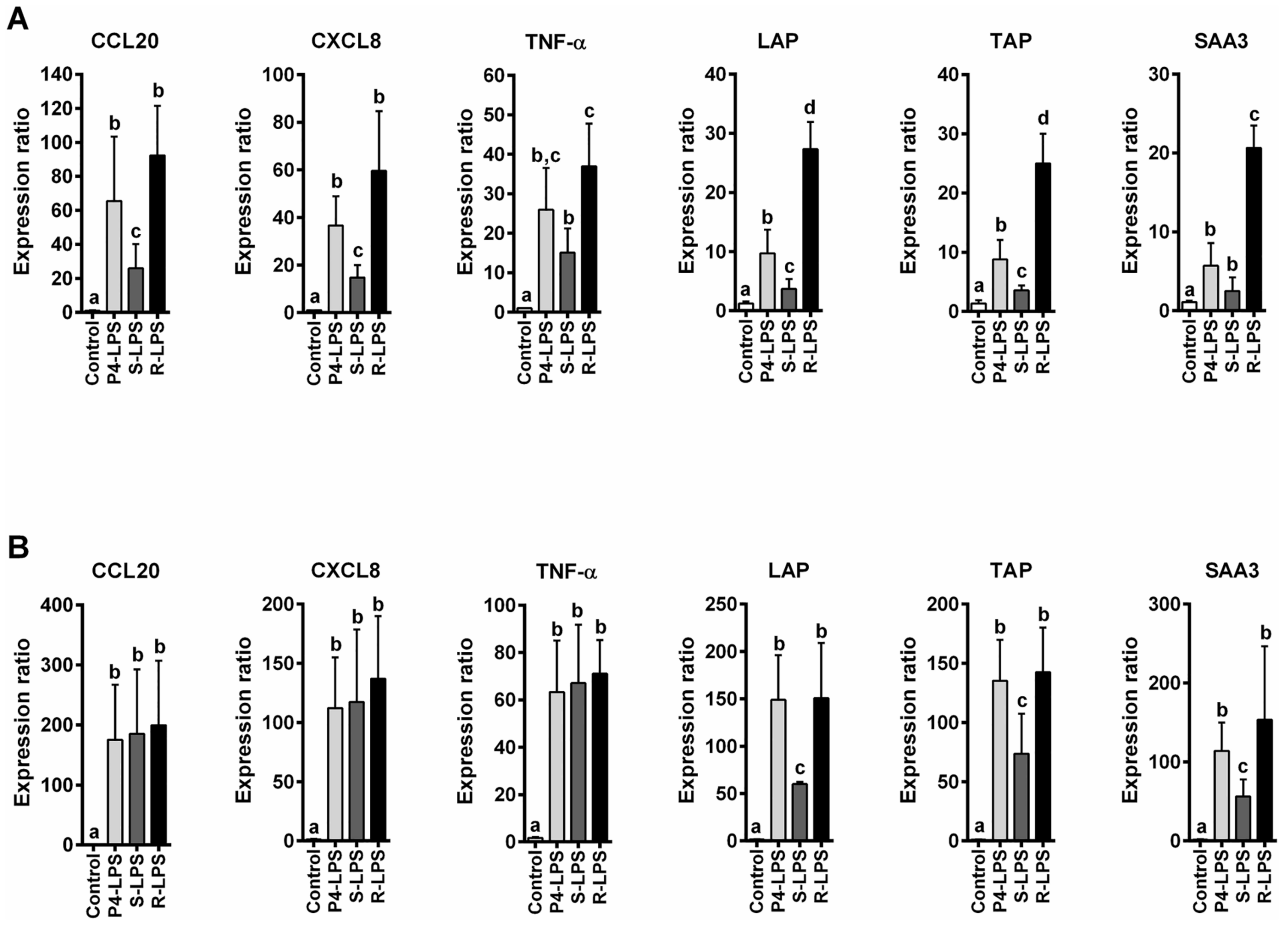
RT-qPCR analysis of the response of PS cells to stimulation by native LPS, S-LPS and R-LPS. PS cells were incubated for 3 hours with 250 ng/mL of native P4 strain LPS (light grey bars), S-LPS (dark grey bars) or R-LPS (dark bars) in stimulation medium in the absence of rbCD14 (A) or in the presence of 0.5 μg/mL rbCD14 (B). Cells were then washed twice with HBSS and medium with gentamicin was added. RNA was extracted 5 hours after beginning of the experiment. Responses were analysed by RT-qPCR and values were normalized against expression of RNA18S, PPIA and ACTB as described previously [36]. Data presented are mean values and standard deviations obtained from three independent experiments. Values with different letters are statistically different from the mock-treated cells and from each other (P < 0.05). p-values were calculated using Mann and Whitney after global comparison using a Kruskal and Wallis test.

To get a more detailed view of the response of PS cells to LPS fractions, the expression of different genes of cytokines, chemokines or defensins was investigated (Fig 7). In the absence of rbCD14, an increased expression of CCL20, CXCL8, TNF-α, LAP, TAP and SAA3 genes was detected in response to R-LPS compared to S-LPS. In general, the response triggered by S-LPS was lower than that observed with native LPS (Fig 7A). The addition of rbCD14 in the medium nullified the difference of expression of genes CCL20, CXCL8 and TNF-α after S-, R- or native LPS treatments. However, an increased expression of TAP, LAP and SAA3 genes was still detected in response of R- or native LPS compared to S-LPS (Fig 7B).

### Magnitude of cytokines, chemokines and CD14 elicitation after intramammary challenge of lactating cows with LPS fractions

**Fig 8:**
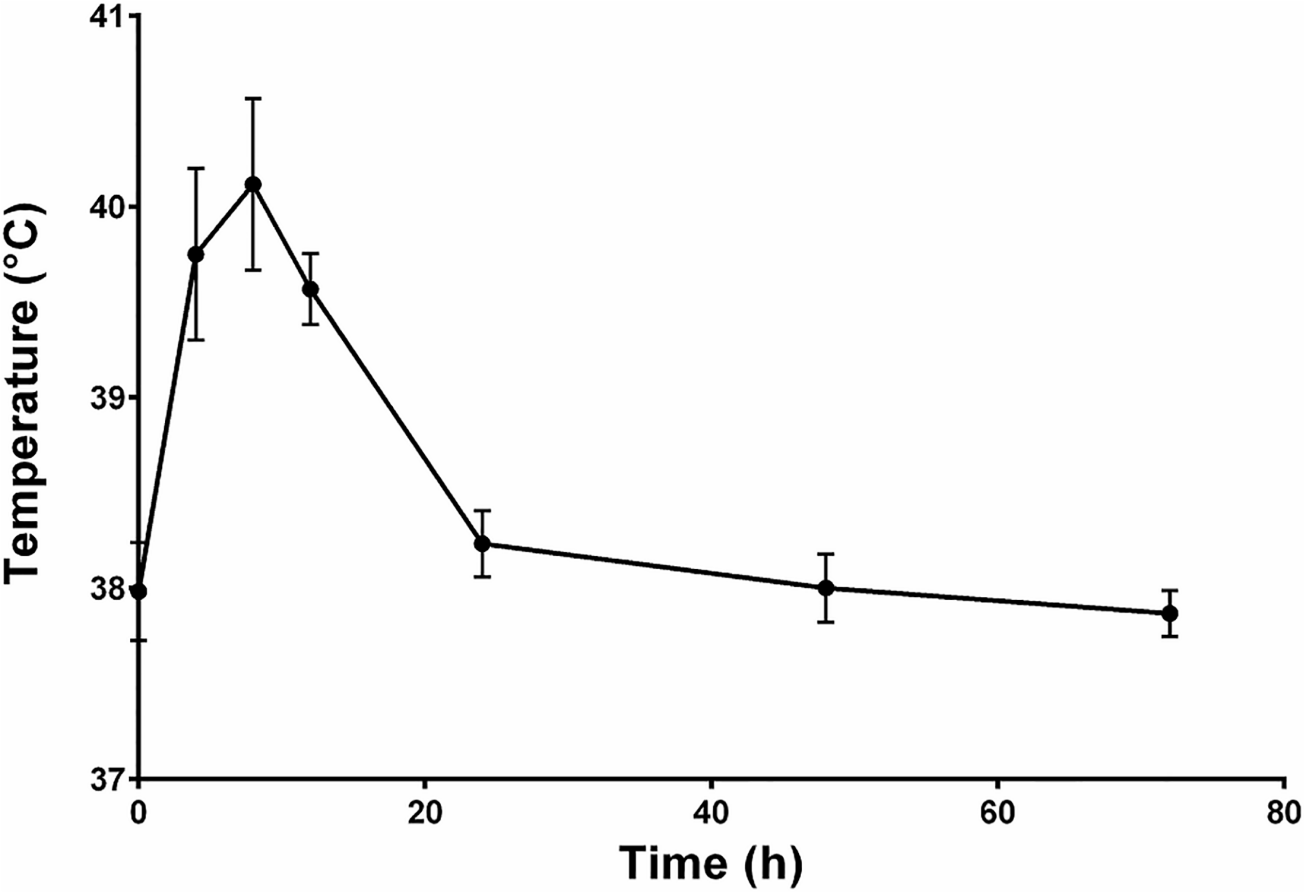
Temperature of cows infused with purified agonists: Native LPS, S-LPS and R-LPS. Each quarter of the udder of six different cows was inoculated with 1μg of native LPS, 1μg of S-LPS; 1μg of R-LPS or an equal volume of PBS-BSA 0.5% in the control quarter. Rectal temperature was measured at 8, 12, 24, 48 and 72 hours post-infusion. Data presented are mean values and standard deviations.

**Fig 9:**
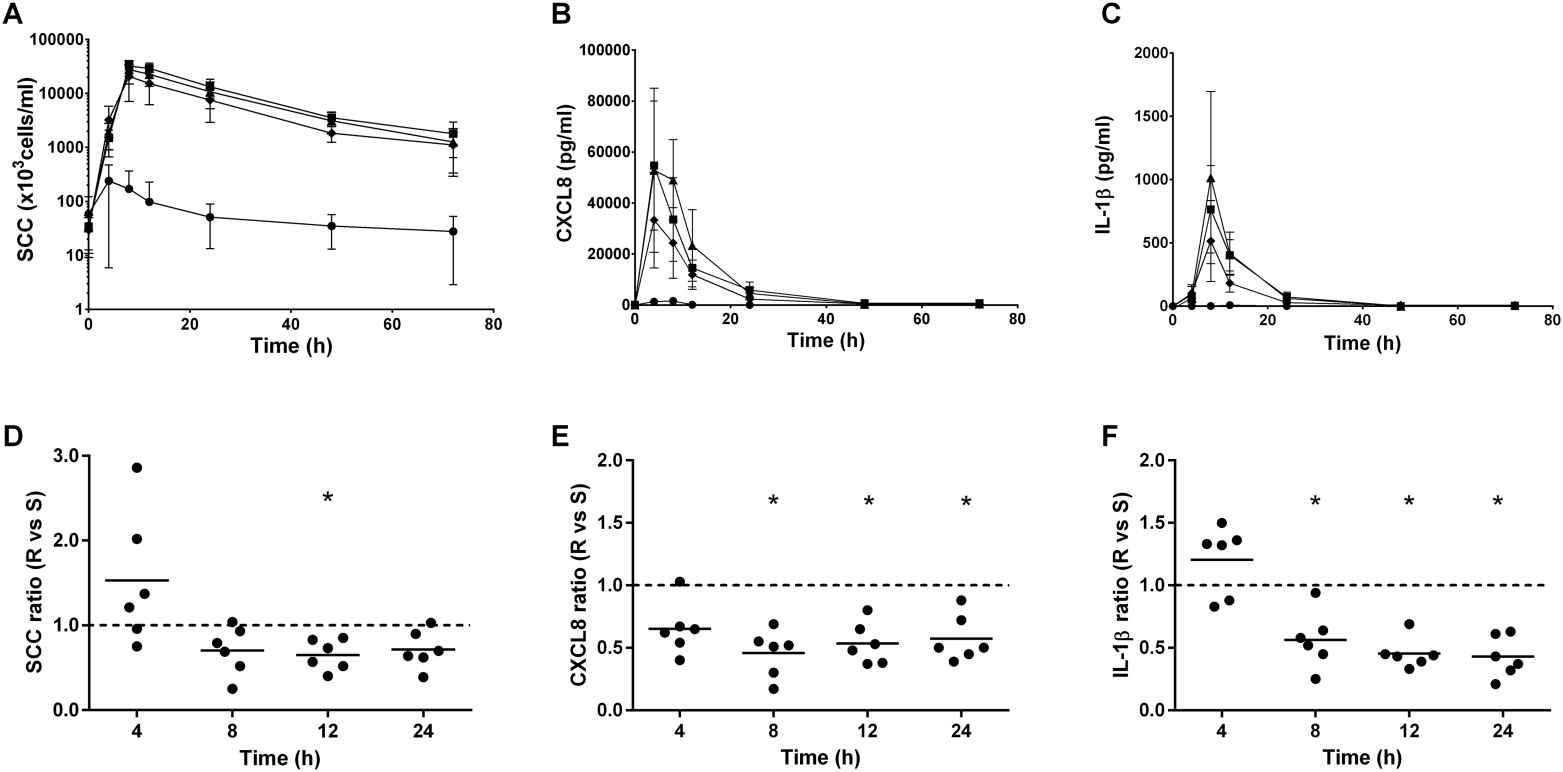
SCC, cytokines and chemokines concentration in milk from quarters infused with purified agonists. Native LPS 1μg (■), S-LPS 1μg (▲); R-LPS 1μg (♦) or an equal volume of PBS-BSA 0.5%(w/v) in the control quarter (●) were infused into each quarter of the udder of six different cows. Response was analysed by quantification of somatic cell count (SCC) (A), CXCL8 (B), IL-1β (C) secretion in milk by ELISA 4, 8, 12, 24, 48 and 72 hours post-infusion. Data presented are mean values and standard deviations. The respective ratio were calculated by dividing the R-LPS response by the S-LPS response for each animal in D, E, and F. A ratio of 1 indicates that the two forms of LPS induce an equally response *in vivo*. Data presented are values from each individual animal with medians. * indicates statistical significance (P < 0.05). P-values were calculated using a Friedmann test.

To evaluate the consequences of the absence of O-antigen on pro-inflammatory responses *in vivo*, the different LPS purified fractions, i.e. native LPS, S- and R-LPS, were infused into three mammary quarters of six cows. In four cows, a control quarter was also infused with DPBS-BSA only. All mock infused quarter appeared free of any inflammatory reaction. As expected, all cows presented a systemic reaction, which was revealed by an increase in rectal temperatures from 38.0°C ± 0.3°C to 40.1 ± 0.7°C at 8 hours post-infusion that was maximal at this time (Fig 8). The inflammatory response was measured by the determination of somatic cells concentrations in milk (Fig 9A and 9D). The SCC significantly increased at 4 hours after S-, R- or native LPS injection compared to the mock infused quarter, and were maximal at 8 hours (Fig 9A). To more precisely investigate the differences triggered by S and R-LPS *in vivo*, we calculated, for each animal, the ratio of SCC obtained with R-LPS divided by that obtained with S-LPS. These ratio showed that the SCC after R-LPS infusion was significantly decreased compared with the S-LPS response at 12 hours after LPS injection (Fig 9D).

To further investigate the association of LPS type with the magnitude of inflammatory reaction, secretion in milk of pro-inflammatory chemokines CXCL8 and CCL20 and cytokines IL-1β and IL-6 were analysed in milk from infused quarter. All of these effectors, including CCL20 whose secretion in milk during inflammation is shown for the first time, were significantly increased after LPS infusion in comparison to mock infused quarter 4 hours post-infusion (Fig 9B and 9C, S6A and S6B Fig). The induction of these cytokines at 4 hours post-infusion were not significantly different after P4 S- or R-LPS forms infusion (Fig 9E and 9F, S6C and S6D Fig). Nevertheless, after 8 hours the induction of CXCL8 and IL-1β after R-LPS infusion was significantly decreased compared with the S-LPS response (Fig 9E and 9F). As well, IL-6 was decreased at 12 hours post infusion (S6C Fig) and CCL20 at 24 hours post infusion after R-LPS challenge compared to S-LPS infusion (S6D Fig).

**Fig 10:**
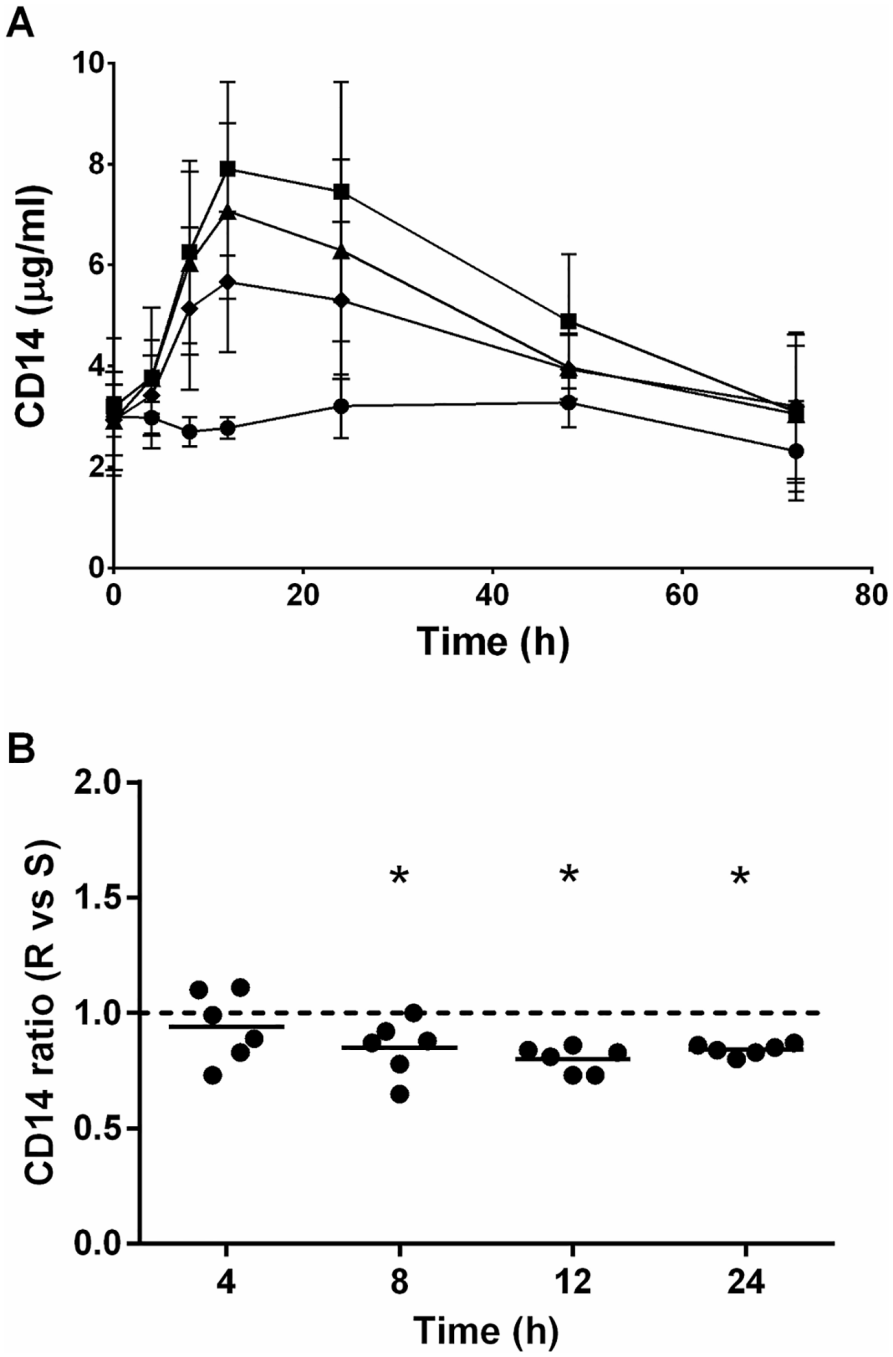
CD14 concentration in milk from quarters infused with purified agonists. Native LPS 1μg (■), S-LPS 1μg (▲); R-LPS 1μg (♦) or an equal volume of PBS-BSA 0.5% in the control quarter (●) were infused into each quarter of the udder of six different cows. Response was analysed by quantification of CD14 secretion in milk by ELISA 4, 8, 12, 24, 48 and 72 hours post-infusion in A. Data presented are mean values and standard deviations. The ratio was calculated by dividing the R-LPS response by the S-LPS response for each animal in B. A ratio of 1 indicates that the two forms of LPS induce an equally response *in vivo*. Data presented are values from each individual animal with medians. * indicates statistical significance (P < 0.05). P-values were calculated using a Friedmann test.

Because rbCD14 had significantly increased *in vitro* the secretion of CXCL8 (Fig 6) and the expression of pro-inflammatory genes after S-LPS treatments on MEC (Fig 7), we quantified precisely the amount of CD14 in milk. Concentrations of CD14 were already detectable before infusion of LPS in the quarter (at the beginning of the experiment) with a CD14 concentration of 3.01μg/mL ± 0.9μg/mL (Fig 10A). Concentration of CD14 was significantly increased after LPS infusion in comparison to mock infused quarter 8 hours after infusion, and was maximal at 12 hours. After R-LPS infusion, CD14 was slightly decreased after 8 hours compared to S-LPS challenge (Fig 10B).

## Discussion

By screening of a library of random transposome mutants of *E*. *coli* P4, we demonstrate that the presence of an O-antigen decreases the ability of this strain to stimulate a pro-inflammatory response by MEC (Fig 1). This was confirmed by comparing the response triggered by the MG1655 K-12 strain that does not have any O-antigen to that of MG1655 *wbbL*+ in which O-antigen synthesis had been restored (S1 Fig) [35]. The lipid A fraction of LPS is considered to be responsible for the pro-inflammatory activity. Yet, as suggested by our results, the role of the O-antigen in this activity should not be overlooked.

Despite the important alterations of the LPS observed in mutants compared to the P4 strain, HEK/TLR4 cells responded in a similar fashion to stimulation with all mutants and WT strain (S2 Fig). A likely explanation is the overexpression in HEK-TLR4 cells of human TLR4 along with co-receptors CD-14 and MD2 that contribute to the recognition of LPS.

Our initial intent was to test whether an increased ability to stimulate mammary epithelial cells would have any consequences on the severity of mastitis. Yet, because alterations observed in our mutants were likely to affect other phenotypes important for the bacteria to trigger mastitis, we first decided to check the consequences of the identified mutations on phenotypes such as the ability to grow in milk, and to resist complement-mediated killing or antimicrobial peptides. Our results show that the absence of O antigen in strain P4, in addition to increasing the innate response of MEC PS cells, has an impact on various phenotypes such as failure to grow in inflammatory milk (Fig 3C) and susceptibility to complement (Fig 4) and polymyxin B (S4 Fig). If any decrease in severity was to be observed using these mutants in a mastitis model, we would be unable to decipher whether such a decrease would only be due to increased ability to trigger the innate immune response of the host or to other phenotypes. We thus decided not to perform these *in vivo* infection experiments.

The impact of the loss of the LPS O-antigen part on the pro-inflammatory response of MEC led us to perform a more detailed analysis. By using purified smooth and rough fractions of LPS, we showed that MEC responded differently to both fractions. Taking advantage of the possibility to supplement or not our cell cultures with rbCD14, we were able to show that the responses to these fractions were modulated by soluble CD14, emphasizing the key role this molecule plays in the recognition of *E*. *coli* by MEC.

Irrespective of the presence of rbCD14 in the medium, the R-LPS fraction induced a stronger response from the PS cells than did the S-LPS fraction as demonstrated by increased CXCL8 secretion (Fig 6). RT-qPCR analysis of the expression of key components of the host innate immune response to infection, such as the chemokines/cytokines CCL20, CXCL8, TNF-α, the two β-defensins, LAP and TAP and the acute phase protein SAA3 [37–44], confirmed the different abilities of R- and S-LPS to stimulate PS cells (Fig 7A). Nevertheless, the addition of CD14 in the media increased responses of MEC to R-LPS but also, and more intensely, to S-LPS (Fig 7B). More specifically, although the addition of rbCD14 in the medium nullified the difference of expression of genes CCL20, CXCL8 and TNF-α after S- or R-LPS treatments, the transcriptomic response of MEC to R and S-LPS fractions were still different as shown by differences in the expression of LAP, TAP and SAA3. As a whole, our results indicate that MEC respond differently to R and S-forms of LPS and that, most interestingly, compared to R-LPS, S-LPS is a much less potent stimulator of the pro-inflammatory response of MEC which requires rbCD14 for efficient stimulation of PS cells (Fig 6).

Deciphering the precise mechanisms and signalling pathways underlying the differences in response to S- and R-LPS will require further investigations.

Indeed, only few studies have focused on purified rough and purified smooth fractions of LPS [45, 46]. Pupo *et al*. have demonstrated that the R- and S- LPS fractions, separated from *E*. *coli* O111 LPS mixture, were highly active at inducing TNF-α in human macrophages in the presence of human serum and that the R-LPS was more active than was the S-LPS fraction [47]. A role for CD14 in the recognition of S-LPS has been demonstrated for other cell types such as murine mast cells which were found to respond normally to R-LPS but did not respond to S-LPS in the absence or at physiological serum concentrations of CD14 [46]. So far, the ability of CD14 to modulate the response to LPS from rough or smooth *E*. *coli* strains had only been demonstrated in myeloid cells [48, 49]. The results presented herein are, to the best of our knowledge, the first ones to demonstrate the contribution of soluble CD14 to the sensing of rough and smooth forms of LPS by epithelial cells.

It is possible that R- and S-LPS fractions, depending on the presence of recombinant bovine CD14, trigger different signalling pathways and regulate different sets of genes, as was observed in our RT-qPCR analyses (Fig 7). As a matter of fact, *in vivo* studies have demonstrated the importance of CD14 in the recognition of LPS in mice indicating that CD14 was required for a TRIF-TRAM mediated response to LPS from a smooth strain [48]. This study by Jiang *et al*. showed that CD14 was required to activate the TRAM-TRIF pathway by both R- and S-LPS while activation of the Myd88 pathway by R-LPS did not require CD14. In addition, activation of the Myd88-independent pathway by LPS was demonstrated in MEC [38]. One could therefore speculate that activation of the Myd88-dependent and TRAM-TRIF-dependent pathways in MEC depends on the type of LPS and the presence of CD14.

It will therefore be of particular interest to investigate how, in MEC, CD14 differentially modulates the two different pathways, i.e. the Myd88-dependent pathway and the TRAM-TRIF pathway, known to be activated upon recognition of LPS [38, 48, 50, 51].

The differences in stimulating capacity of R- and S-LPS is not restricted to *E*. *coli*. Huber *et al*. previously found that an R-form fraction isolated from *Salmonella* Minnesota mutant exhibited much higher biologic activity compared to the parental LPS in inducing higher IL-6 from murine mast cells and macrophages. The *in vitro* data were corroborated by an *in vivo* study measuring TNF-α level in plasma, in response to injection of R- and S- LPS fractions in mice [46]. Two mutants in LPS biosynthesis genes, *wlbA* and *wlbL*, of *Bordetella avium* which presented a rough phenotype, were affected in their ability to colonize turkey trachea. In addition, the authors showed that the biosynthesis of a full-length LPS molecule by three other species of *Bordetella*, *B*. *pertussis*, *B*. *parapertussis*, and *B*. *bronchiseptica*, is essential for the expression of full virulence in mice [52].

Our results suggest that the absence of O antigen could modify both the extent and the type of the innate immune response of MEC. Thereby, the immune response of the udder could vary depending on the type of LPS. An intramammary challenge with R- or S-LPS fractions of *E*. *coli* P4 in cows both induced an inflammation but some differences were observed. R- and S-LPS induced the same recruitment of neutrophils with comparable cytokines IL-6, IL-1β and chemokines CXCL8 and CCL20 magnitude at 4 hours post-infusion. However, production of CXCL8 and IL-1β after R-LPS infusion was significantly decreased compared with the S-LPS response after 8 hours (Fig 9). Additionally, IL-6 was decreased at 12 hours post infusion and CCL20 at 24 hours post infusion after R-LPS challenge compared to S-LPS infusion (S6 Fig). The two forms of LPS trigger the same early immune response at the beginning of the inflammation, but after 8 hours the response induced by S-LPS is more intense. The presence of O antigen induces a more potent innate response in the udder.

We also confirmed that the concentrations of sCD14 in fresh aseptic milk were approximately 3 μg/mL. The protective effect of recombinant bovine CD14 against infection by *E*. *coli* was demonstrated in a mouse mastitis model [53]. Furthermore, the addition of recombinant bovine CD14 to udder quarters inoculated with *E*. *coli* significantly increased somatic cell counts in milk and reduced bacterial loads in infected quarter [54]. Huber *et al*., have demonstrated in mouse that similar concentrations enabled CD14-negative cells to respond to S-form LPS. Indeed, these high concentrations of sCD14 enhanced the IL-6 response of mast cells to S-form LPS [46]. This suggests that the up-regulation of sCD14 in the course of the inflammatory response, observed in our *in vivo* analysis, ensures the contribution of other CD14-negative LPS target cells to the defence against S-LPS. The presence of sCD14 in milk could facilitate the sensing and the response of MEC or CD14-negative cells to S-LPS and so counteract the potential anti-inflammatory effect of O-chain. Thus, S-LPS could lead to an early stronger stimulating action of immune system as well as R-LPS. Moreover, during *in vivo* experiments membrane anchored-CD14 of myeloid cells present in milk or mammary tissues could also amplify the stronger response of immune system.

In conclusion, our studies with *E*. *coli* P4 provide a first line of experimental evidence that the LPS O-antigen moiety is associated with the cytokine production by MEC and show that R-LPS have a higher ability to activate MEC compared to S-LPS. However, *in vivo*, under physiological conditions with high concentrations of rbCD14 in milk, the S-LPS has the potential to stimulate the innate immune response.

Recently, Horvath *et al*. provided experimental evidence that the magnitude of cytokine elicitation was correlated with LPS serotype during urinary tract infection [16]. Despite the lack of association of particular O-serotypes with *E*. *coli* isolated from mastitis cases [55, 56], our results with K-12 *wbbL*+ (serotype O16) and P4 (serotype O32) indicated that the structure of the O-antigen may have an impact on the response of MEC. Indeed, in absence of CD14, K-12 *wbbL*+ is not recognized by MEC whereas P4 induces a weak stimulation by MEC. Variation in O-antigen sugar structure may thus determine the strength of cytokines induction by MEC. Future studies should include evaluation of a repertoire of LPS serotypes of mastitis *E*. *coli* to further analyse how the LPS O-antigen moiety is associated with cytokines production by bovine mammary epithelial cells and infection clinical features.

## Supporting information

S1 Fig CXCL8 secretion by PS cells upon stimulation with live *E*. *coli* K-12 MG1655 wild type (Rough) and its derived strain MG1655 *wbbL*+ (Smooth). PS cells were incubated for 3 hours with the indicated strains at a MOI of 1 in stimulation medium in the absence of rbCD14 (white bars) or in the presence of 0.5 μg/mL rbCD14 (grey bars). Cells were then washed twice with HBSS and medium with gentamicin was added. Response was analysed by quantification of CXCL8 secretion by ELISA in supernatants collected 8 hours after beginning of the stimulation. Data presented are mean values and standard deviations obtained from three independent experiments with stimulations performed in duplicates. * indicates statistical significance (p < 0,05). p-values were calculated using Mann and Whitney after global comparison using a Kruskal and Wallis test.
(TIF)

S2 Fig CXCL8 secretion by HEK/TLR4 cells upon stimulation with live *E*. *coli* P4 wild type and mutants. Cells were incubated for 3 hours with the indicated strains at a MOI of 1. Cells were then delicately washed once with HBSS and medium with gentamicin was added. Response was analysed by quantification of CXCL8 secretion by ELISA 8 hours after beginning of the experiment. Data presented are mean values and SD obtained from three independent experiments with stimulations performed in duplicates. p-values were calculated using Mann and Whitney after global comparison using a Kruskal and Wallis test.
(TIF)

S3 Fig CXCL8 secretion by HEK/TLR2 cells upon stimulation with live *E*. *coli* P4 wild type and mutants. Cells were incubated for 3 hours with the indicated strains at a MOI of 1. Cells were then delicately washed once with HBSS and medium with gentamicin was added. Response was analysed by quantification of CXCL8 secretion by ELISA 8 hours after beginning of the experiment. Data presented are mean values and SD obtained from three independent experiments with stimulations performed in duplicates. p-values were calculated using Mann and Whitney after global comparison using a Kruskal and Wallis test.
(TIF)

S4 Fig Polymyxin B susceptibility of *E*. *coli* P4 wild type and mutants. Broth microdilutions were performed for determination of polymyxin B MICs in Mueller-Hinton broth. A final concentration of 2.10^6^CFU/mL in each well was inoculated. Polymyxin B was used at the concentration range from 0.0625 to 64 μg/mL. Panels were incubated 24 hours at 37°C. The MIC was considered the lowest drug concentration inhibiting growth. Data presented are mean values and SD obtained from four independent experiments. * indicates statistical significance (p < 0,05). p-values were calculated using Mann and Whitney after global comparison using a Kruskal and Wallis test.
(TIF)

S5 Fig TLR2 activity of purified LPS. HEK293 cells stably expressing TLR2 were stimulated with LPS from the *E*. *coli* P4 natural preparation and fractions derived thereof (Smooth-fraction, S-LPS fraction and Rough-fraction, R-LPS fraction) or with the synthetic lipopeptide Pam3CSK4 at the indicated concentrations. Unstimulated cells were included as negative controls. After 24 h, TLR2 activation was measured by detection of secreted alkaline phosphatase. Data shown is one experiment representative of three independent experiments.
(TIF)

S6 Fig IL-6 and CCL20 concentration in milk from quarters infused with purified agonists. Native LPS 1μg (■), S-LPS 1μg (▲); R-LPS 1μg (♦) or an equal volume of PBS-BSA 0.5% in the control quarter (●) were infused into each quarter of the udder of six different cows. Response was analysed by quantification IL-6 (A) and CCL20 (B) secretion in milk by ELISA 4, 8, 12, 24, 48 and 72 hours post-infusion. Data presented are mean values and standard deviations. The respective ratio were calculated by dividing the R-LPS response by the S-LPS response for each animal in C and D. A ratio of 1 indicates that the two forms of LPS induce an equally response *in vivo*. Data presented are values from each individual animal with medians. * indicates statistical significance (P < 0.05). P-values were calculated using a Friedmann test.
(TIF)

S1 Table List of primers used for RT-qPCR. (DOCX)

S1 Dataset Raw data. (XLSX)
