# Supplementary figures and images for: Sensing of Escherichia coli and LPS by mammary epithelial cells is modulated by O-antigen chain and CD14
Source: PLoS One. 2018 Aug 24;13(8):e0202664. doi: 10.1371/journal.pone.0202664 (PMC6108492; doi:10.1371/journal.pone.0202664)

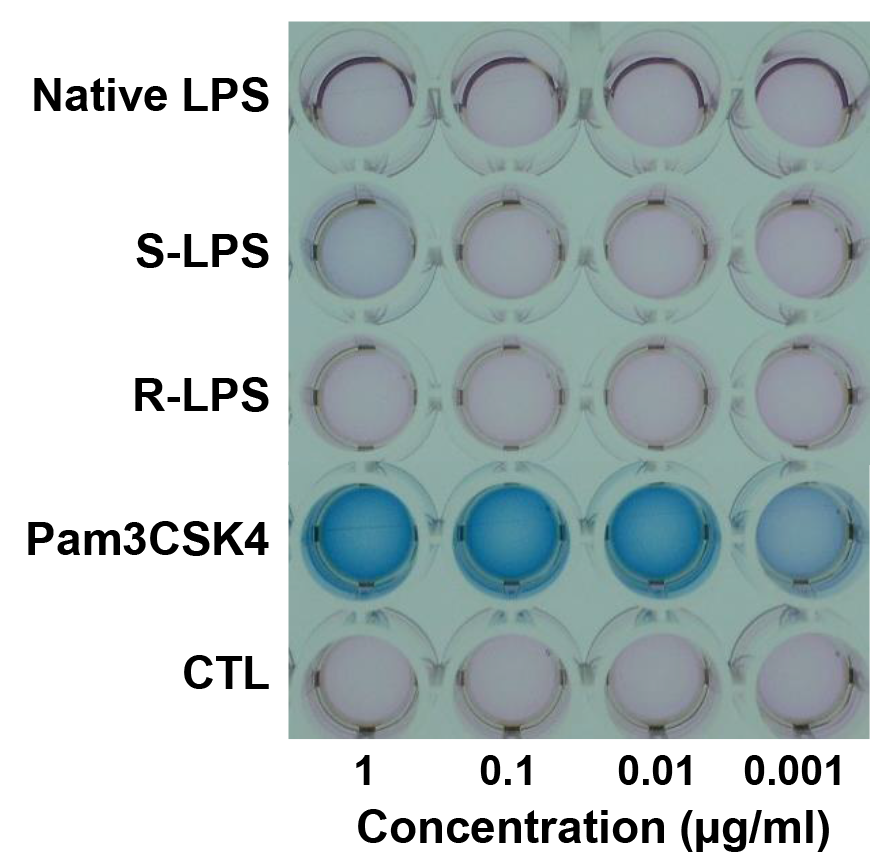

Supplement: S5 Fig — HEK293 cells stably expressing TLR2 were stimulated with LPS from the E. coli P4 natural preparation and fractions derived thereof (Smooth-fraction, S-LPS fraction and Rough-fraction, R-LPS fraction) or with the synthetic lipopeptide Pam3CSK4 at the indicated concentrations. Unstimulated cells were included as negative controls. After 24 h, TLR2 activation was measured by detection of secreted alkaline phosphatase. Data shown is one experiment representative of three independent experiments. (TIF) [file pone.0202664.s005.tif]

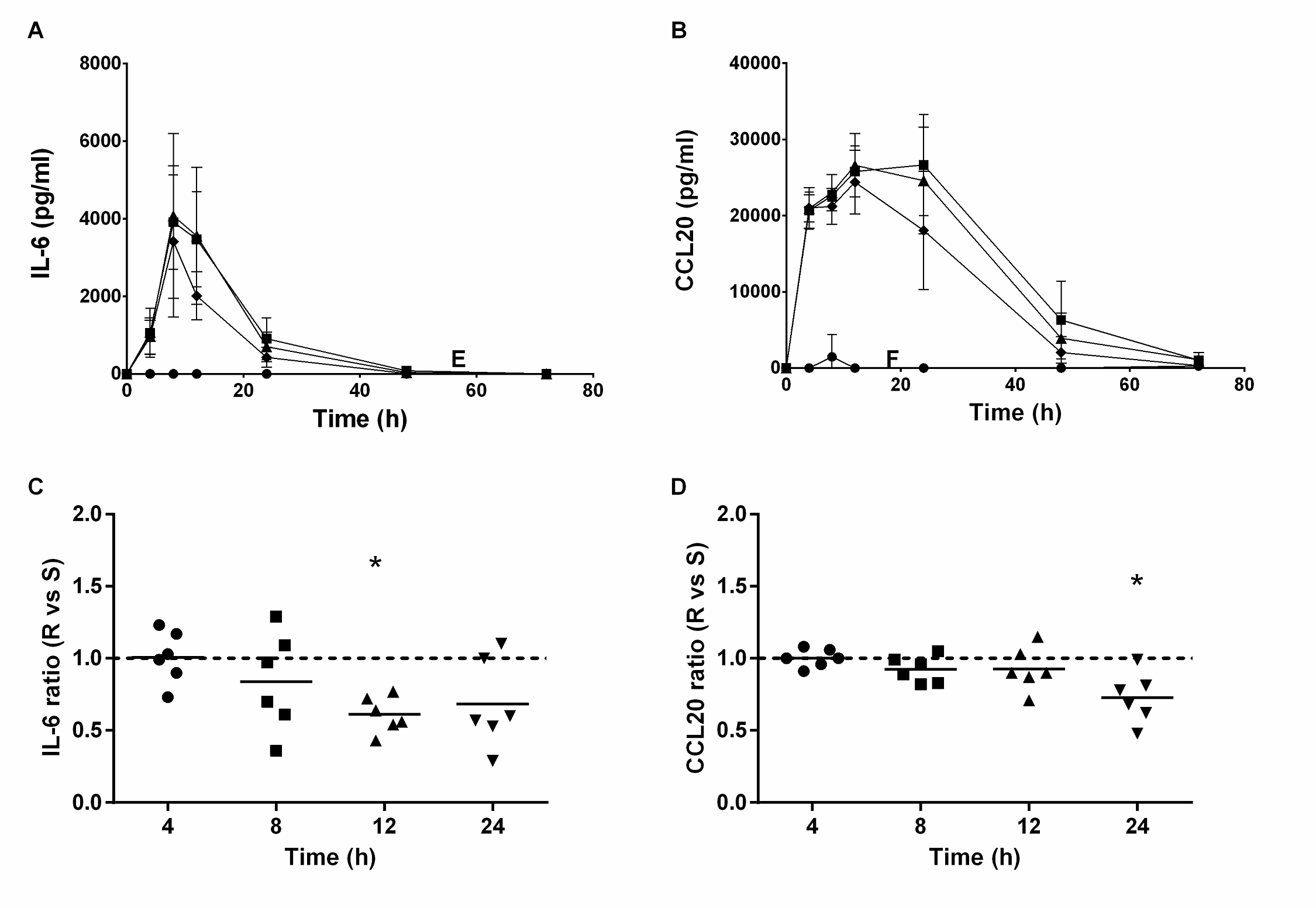

Supplement: S6 Fig — Native LPS 1μg (■), S-LPS 1μg (▲); R-LPS 1μg (♦) or an equal volume of PBS-BSA 0.5% in the control quarter (●) were infused into each quarter of the udder of six different cows. Response was analysed by quantification IL-6 (A) and CCL20 (B) secretion in milk by ELISA 4, 8, 12, 24, 48 and 72 hours post-infusion. Data presented are mean values and standard deviations. The respective ratio were calculated by dividing the R-LPS response by the S-LPS response for each animal in C and D. A ratio of 1 indicates that the two forms of LPS induce an equally response in vivo. Data presented are values from each individual animal with medians. * indicates statistical significance (P < 0.05). P-values were calculated using a Friedmann test. (TIF) [file pone.0202664.s006.tif]
