# Supplementary material for: Sensing of Escherichia coli and LPS by mammary epithelial cells is modulated by O-antigen chain and CD14
Source: PLoS One. 2018 Aug 24;13(8):e0202664. doi: 10.1371/journal.pone.0202664 (PMC6108492; doi:10.1371/journal.pone.0202664)
Supplement: S1 Table — (DOCX) [file pone.0202664.s007.docx]

| **Gene** | **Oligonucleotides (5'-3') F: forward ; R: reverse** | **Amplicon (pb)** | **Annealing  temperature (°C)** |
| --- | --- | --- | --- |
| 18S RNA | F: CGGGGAGGTAGTGACGAAA | 196 | 62 |
|  | R: CCGCTCCCAAGATCCAACTA |  |  |
| ACTB | F: ACGGGCAGGTCATCACCATC | 166 | 64 |
|  | R: AGCACCGTGTTGGCGTAGAG |  |  |
| PPIA | F: TCCGGGATTTATGTGCCAGGG | 206 | 65 |
|  | R: GCTTGCCATCCAACCACTCAG |  |  |
| CCL20 | F: TTCGACTGCTGTCTCCGATA | 172 | 62 |
|  | R: GCACAACTTGTTTCACCCACT |  |  |
| CXCL8 | F: TGAAGCTGCAGTTCTGTCAAG | 202 | 62 |
|  | R: TTCTGCACCCACTTTTCCTTGG |  |  |
| LAP | F: TGCTCCTTGGGCTCCTCTTC | 149 | 64 |
|  | R: CTCCGAGACAGGTGCCAATC |  |  |
| SAA3 | F: CCTCAAGGAAGCTGGTCAAG | 226 | 62 |
|  | R: TACCTGGTCCCTGGTCATAC |  |  |
| TAP | F: GTAGGAAATCCTGTAAGCTGTG | 139 | 62 |
|  | R: GTGTCTTGGCCTTCTTTTAC |  |  |
| TNF-α | F: TCTTCTCAAGCCTCAAGTAACAAGC | 104 | 62 |
|  | R: CCATGAGGGCATTGGCATAC |  |  |
